# Degradation of Carbon Fiber-Reinforced Polymer Composites in Salt Water and Rapid Evaluation by Electrochemical Impedance Spectroscopy

**DOI:** 10.3390/ma16041676

**Published:** 2023-02-17

**Authors:** Hanlu Zhang, Fabao Kong, Yuchao Dun, Xueping Chen, Quankai Chen, Xuhui Zhao, Yuming Tang, Yu Zuo

**Affiliations:** 1 Key Laboratory of Carbon Fiber and Functional Polymers, Ministry of Education, Beijing University of Chemical Technology, Beijing 100029, China; 2 Corrosion Protection and Materials Research Laboratory, No. 92228 of the People’s Liberation Army, Beijing 100072, China; 3 Aviation Key Laboratory of Science and Technology on Advanced Surface Engineering, AVIC Manufacturing Technology Institute, Beijing 100024, China

**Keywords:** carbon fiber-reinforced composite, vinyl resin, bismaleimide resin, EIS, evaluation

## Abstract

The electrochemical impedance spectroscopy and weight gain tests were performed on carbon fiber/vinyl ester and carbon fiber/bismaleimide composites in 3.5% NaCl solution to study the electrochemical and water absorption behaviors. The microstructure morphology and the flexural property of the composites in the long-term exposure process were analyzed with the scanning electron microscope and four-point bending tests. The results revealed that after long-time immersion (>200 d), the water absorption of the two composites is less than 0.5%. This has little effect on the microstructural integrity, only with slight damage on the fiber/resin interfaces, but results in a significant decrease (about 84%) in the composite flexural property. The variation of the water absorption percentage shows good consistency with that of the resin capacitance (*Q*_c_) and is negatively related to the variation of the resin resistance (*R*_po_) and the low-frequency impedance (|*Z*|_0.01Hz_) of the composites. A good linear relationship exists between the variations of phase angles in the middle-frequency range (0.1−10 Hz) and the |*Z*|_0.01Hz_. The phase angle at 10 Hz (*θ*_10Hz_) may be suggested as a suitable parameter to rapidly evaluate the performance of carbon fiber-reinforced polymer composites, just like for evaluating the protective performance of polymer-coated metals in the literature.

## 1. Introduction

Because of their high specific strength, high stiffness, and very good corrosion resistance, carbon fiber reinforced polymers (CFRP) have been widely used in aircraft, aerospace, automotive, civil, and electronics industries. Compared with metallic engineering materials, carbon fiber-reinforced composites have much better corrosion resistance. However, long-time immersion in aggressive service environments or galvanic coupling with engineering metals (steels and aluminum alloys, etc.) would lead to a decrease in the stability or even accelerate the degradation of the composites [1,2,3,4,5].

Carbon fiber/vinyl ester and carbon fiber/epoxy systems are often chosen because of their easy processing and potential durability considerations [6]. Compared with epoxy resin, vinyl ester resin has a relatively low cost and superior chemical stability in salt water, so more and more vinyl ester-based carbon-reinforced composites have been used as structural materials in marine environments [6,7,8]. Marouani et al. studied the durability of carbon fiber-reinforced composites with epoxy and vinyl ester matrixes in two kinds of aging conditions [6]. Their research results showed that vinyl ester composites have better hydrolysis resistance, even at elevated temperatures. Kootsookos et al. comparatively studied the chemical stability of vinyl ester-based and polyester-based carbon fiber composites [7]. They concluded that with the amounts of water absorption increasing, some chemical degradation occurs to the vinyl ester-based composite, but to a much lower extent than that for the polyester-based carbon fiber composite. Afshar et al. studied the flexural property variation of the carbon fiber vinyl ester composite in indoor and outdoor conditions and discussed the correlation between long-term outdoor exposure and laboratory conditions [8]. Alias et al. investigated the damage to the carbon fiber vinyl ester composite from cathodic polarization and concluded that two types of damage, blistering and dissolution, occurred because of the galvanic interactions and seawater immersion [9].

Bismaleimide (BMI) resin matrix carbon fiber reinforced composites possess very good thermal stability and mechanical properties; thus, they are currently being used as structural materials for military and civilian aircraft [10,11]. Akay et al. studied the effects of long-term exposure at high temperatures on the interlaminar shear and impact performance of a carbon fiber bismaleimide composite [11]. Bao et al. studied the moisture absorption and hygrothermal aging of a bismaleimide resin-based carbon fiber composite material, and the results indicated that the composites present a two-stage diffusion behavior [12]. Li et al. investigated the mechanical properties of T700 carbon fiber-reinforced bismaleimide composites under hygrothermal conditions and discussed the synergistic effect of moisture absorption and temperature on the decrease in mechanical properties [13].

In carbon fiber-reinforced polymer composites, the integrity of the interface between the carbon fiber and the matrix is usually evaluated macroscopically via the interlaminar shear strength (ILSS) or the interfacial shear strength (IFSS) [4]. However, these are destructive methods and are not suitable for the long-term monitoring of composite properties. Electrochemical impedance spectroscopy (EIS) is a non-destructive electrochemical technique that has been widely used in the field of polymeric coatings for studying the coating degradation and monitoring the corrosion process at the metal-coating interface. Through spectra analysis or the use of equivalent circuit modeling, the delamination of the coating from the substrate can be quantitatively measured, and the coating failure can be predicted. Since carbon fibers possess electrical conductivity, the EIS technique could be applied to study the property variation of carbon fiber-reinforced composites [9,14]. Alias et al. and Kaushik et al. measured the impedance spectra of carbon fiber/vinyl ester composite and carbon fiber/epoxy composite in 3.5% NaCl solution and analyzed the impedance data by the equivalent circuit models, which are commonly used for coated metals study [15,16]. It was suggested that the resistance of polymer matrix (*R*_po_) determined from the circuit models might offer a damage-monitoring method for carbon fiber composite materials. Taylor et al. studied the impedance spectra of a graphite fiber/bismaleimide composite under conditions of applied polarization and coupled with metals in 0.6 M NaCl solution [17]. They suggested that EIS might be a sensitive method for monitoring the changes in surface morphology and chemistry of carbon fiber reinforced composites, especially since the phase angle is very sensitive in characterizing the changes in the composite. Mueller et al. investigated the damage behavior of carbon fiber-reinforced polyetheretherketone composites coupled with titanium and stainless steel in a simulated body fluid by polarization and EIS techniques [18]. They pointed out that monitoring electrochemical parameters offers the possibility to analyze changes in the properties of carbon fiber composites. Zhang et al. studied the electrochemical behaviors of carbon fiber-reinforced polymers with epoxy and nylon matrix in simulated auto industry solution by EIS method and analyzed the influence of the microstructures and defects in the surface polymer layers on the electrochemical performance of the composites [19,20].

The conventional EIS analysis is based on the analysis of the impedance spectra to obtain the impedance parameters which can reflect the performance of the coating. It is tedious work and not suitable for field tests. The impedance modulus at low-frequency (|*Z*|_0.01Hz_) is generally recognized for estimating the protectiveness of polymer coatings. Usually, the higher the value of |*Z*|_0.01Hz_, the better the protection performance of the polymer coatings possess [21]. In order to quickly evaluate the coating performance, some investigators suggested several methods based on the EIS technique, which could avoid fitting and analyzing complex impedance spectra to some extent [22,23,24,25]. Among them, the breakpoint frequency (*f*_b_) method proposed by Haruyama et al. has attracted the attention of many investigators [22], where *f*_b_ is the frequency at which the phase angle equals 45°, which can be used to estimate the extent of delamination area at the interface between coating and metal without the measurement in the low-frequency region. In our previous studies [26,27], the measurement of the phase angles in middle-frequency (such as 10 Hz) was proposed for evaluating the protective performance of organic coatings because the variation of the phase angles in middle-frequency was found to follow a similar trend to that of the low-frequency impedance (|*Z*|_0.01Hz_) for many organic coating systems. Since the measurement of the phase angles in the middle-frequency domain takes a very short time, it is very suitable for rapid testing and evaluation in field applications. Some researchers applied the variation of phase angle at 10 Hz to evaluate the performance of polymer coatings and suggested it can be used as a fast approach for monitoring the degradation process of the polymer coating on a metal substrate [28,29,30,31,32,33]. However, no work has currently been reported about the applicability of the phase angle method in evaluating the performance of carbon fiber-reinforced polymer composites.

In this work, two kinds of carbon fiber-reinforced polymer composites (a carbon fiber/vinyl ester composite and a carbon fiber/bismaleimide composite) were selected to be the research objects. The EIS technique and weight gain test, combined with a four-point bending test and scanning electron microscopy observation, were employed to study the degradation characteristic of the composites during long-time immersion (>200 d) in 3.5% NaCl solution. The impedance parameters and their relationship to the water adsorption behavior of the composites were emphatically analyzed. Based on these, the correlation between variations of the phase angles in the middle-frequency range and the |*Z*|_0.01Hz_ during the degradation process of the composites was discussed. The objective is to provide a rapid evaluation method suitable for monitoring the structural integrity and performance of carbon fiber-reinforced polymer composites.

## 2. Experimental

### 2.1. Materials and Preparation

Because carbon fiber/vinyl ester-reinforced composites (CF/VE) have been widely used as structural materials in marine environments due to their relatively low cost and superior chemical stability, and carbon fiber/bismaleimide-reinforced composites (CF/BMI) have very good thermal stability and mechanical properties. Thus, the CF/VE and CF/BMI composite materials were selected in this work as the study materials; both are unidirectional carbon fiber-reinforced polymer composites.

In the CF/VE composite, a vinyl ester resin (Derakane MOMENTUM 411-350) was the matrix, which was catalyzed and promoted with methylethylketon perosxide and 6% cobalt naphthenate in the amounts of 1 phr and 0.05 phr, respectively, and T700 carbon fibers (Toray, Japan) coated with type FOE sizing was used as reinforcement. The composite was fabricated in the VARTM process at ambient temperature and then post-cured at 120 °C for 2 h. The carbon fiber volume fraction in the composite was about 60%. Some of the cured plates were cut into 50.8 × 50.8 × 1 mm^3^ specimens for weight gain and EIS tests and some 140 × 20 × 1 mm^3^ specimens for a four-point bending test. In the CF/BMI composite, a 5428 bismaleimide resin (developed by Beijing Institute of Aeronautical Materials) with high toughness was the matrix, and T700 carbon fibers (Avic Composite Materials Co. Ltd., Beijing, China) coated with type FOE sizing was reinforcement. The composite was fabricated in the high-temperature resin transfer molding process. The carbon fiber volume fraction in the composite was about 55%. The cured CF/BMI composite plates had the same length and width as CF/VE CF/BMI with a 2 mm thickness. Table 1 shows the composition of these two composite materials.

**Figure 1:**
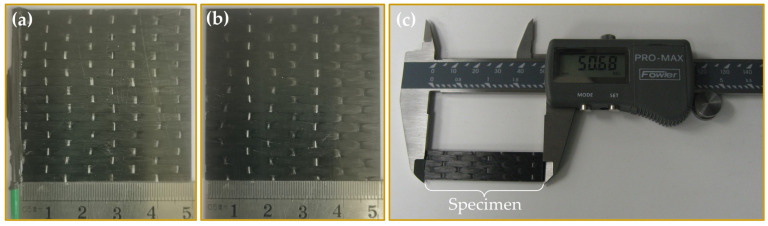
The macrosopic specimens used for (**a**) EIS test; (**b**) weight gain test; (**c**) mechanical property test.

For the EIS test, some specimens were prepared as working electrodes. The preparation method is as follows [34]. One side of the specimen, which was perpendicular to the carbon fibers’ direction, was polished with sandpapers to expose the fibers. Then, an electrical connection was made using a nickel print coating (GC Electronics #22-207) to connect a copper wire to the side of the composite with exposed carbon fiber ends. After 24 h, an epoxy resin was applied to seal and fix the nickel print coating and the copper wire to the specimen. The other three edges of the specimen were also sealed by the epoxy resin and cured for 6 h prior to immersion. Figure 1 presents the macroscopic specimens for the EIS test, weight gain test, and mechanical property test.

### 2.2. Measurements

Specimens were exposed to 3.5% NaCl solution in the open circuit potential state at ambient temperature. The EIS, weight gain, four-point bending, and scanning electronic microscopy (SEM) measurements were carried out periodically for the specimens.

The EIS test was performed using a PARSTAT 2273 instrument (Princeton, NJ, USA). A 5 mV perturbation was applied with a 10^5^ Hz−10^−2^ Hz scanning frequency range, and 30 frequencies were adopted. A three-electrode system was employed, in which the reference electrode was a saturated calomel electrode (SCE), the counter electrode was a platinum wire, and the composite specimen was the working electrode with 10 cm^2^ exposed testing area. The test was carried out at an ambient temperature, and the number of parallel specimens was three. The data were fitted by the Zsimpwin Software (V 3.50).

The weight of the specimens before and after immersion in 3.5% NaCl solution for different periods was measured by an analytical balance (YP2003, Shanghai Yueping Science Instrument, Shanghai, China). The original specimen mass was recorded as *m*_0_. The specimen was withdrawn from the solution at regular intervals, washed and cleaned with deionized water, then carefully wiped dry with a paper towel to remove surface moisture and weighed. The mass of the specimen after a given immersion time was recorded as *m*_t_. The percentage mass change (*M*/%_t_) of the specimen was calculated by Formula (1):

(1) $$M/{\%}_{t}=\frac{m_{t}-m_{0}}{m_{0}}$$

The four-point bending test was performed by an MTS Insight 50 Universal Testing Machine (INSTRON-1121, INSTRON, Norwood, MA, USA) according to ASTM D6272-02 standard. Prior to the test, the specimen was withdrawn from immersion and carefully wiped dry with a paper towel. The applied crosshead speed was 1.5 mm/min. Five specimens were tested for each condition, and the mean value of flexural modulus (*E*_f_) was determined by Formula (2). Where *m* is the gradient of the linear portion of the load–displacement curve, *w* and *h* are specimen width (20 mm) and thickness (1 mm), respectively, and *L* is the support span length (39 mm).

(2) $$E_{f}=\frac{0.17L^{3}m}{wh^{3}}\;(\mathrm{GPa})$$

The morphological structure of the composite specimen was characterized by a scanning electronic microscope (Hitachi S4700, Tokyo, Japan). The chemical element analysis was performed with energy disperse spectroscopy (EDS) (QUANTAX 70, BRUKER, Berlin, Germany). Before the SEM test, the specimen was cleaned, wiped dry, and sprayed with gold powder.

## 3. Results and Discussion

### 3.1. EIS Spectra of T700/VE Composite in 3.5% NaCl Solution

**Figure 2:**
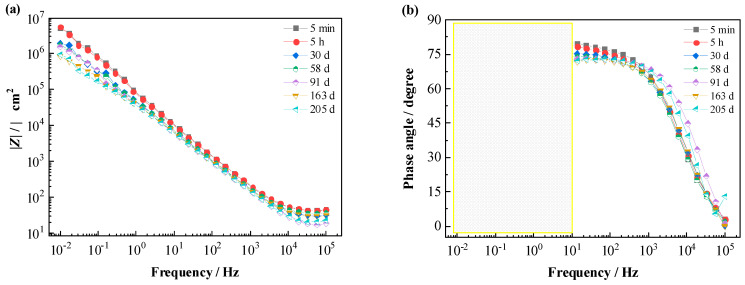
EIS Bode spectra of T700/VE composite in 3.5% NaCl solution. (**a**) Impedance modulus; (**b**) Phase angle.

Figure 2 shows the Bode magnitude and phase angle plots of EIS spectra for the T700/VE composite specimen in 3.5% NaCl solution. After long-time immersion (205 d), there is no big change in the plots. At the start of exposure (5 min), the low-frequency impedance at 0.01 Hz (|*Z*|_0.01Hz_) is 5.0 × 10^6^ Ω cm^2^, and the phase angle values approach 80 degrees in a wide frequency range (<100 Hz), which exhibits that the impedance response is very capacitive and the composite presents little conductivity [17,18]. As exposure progressed, the phase angle values in low and middle frequencies (<10 Hz) decreased slightly, indicating a gradually increasing ionic conductivity of the composite, possibly due to the slow penetration of electrolyte through the polymer matrix [16]. After 205 d, the value of |*Z*|_0.01Hz_ is about 1.0 × 10^6^ Ω cm^2^ with a decrease in less than one order of magnitude compared with the original value, and the phase angle at 0.01 Hz is higher than 54 degrees. These demonstrate that the T700/VE composite has very good chemical stability in salt water at open circuit potential conditions, which verifies the results in the literature [6,7,8].

**Figure 3:**
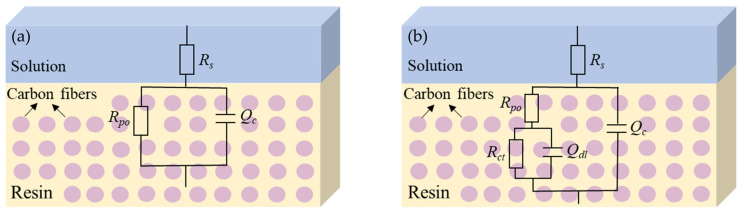
Equivalent circuit models used for EIS data fitting. (**a**) Model A; (**b**) Model B.

The impedance data were fitted using the equivalent circuit models in Figure 3 [15,16]. The variations of the fitting parameters were analyzed. The constant phase angle element (*Q*) was used to simulate the non-ideal capacitance behavior of the composite electrode. In the models, *R*_s_ represents the solution resistance; *Q*_c_ and *R*_po_ represent the information at the interface between polymer and moisture, which is the constant phase angle element and the pore resistance of the matrix polymer due to the penetration of electrolyte from the surface through the defects to the fibers, respectively; *Q*_dl_ and *R*_ct_ represent the impedance behavior of the interface between carbon fibers and moisture, which is the double layer capacitance and the charge transfer resistance related to electrochemical reactions, respectively. Model A was applied in the initial stage (0−39 d). Model B was applied in the data fitting after the electrolyte penetrated the surface of carbon fibers with the time extended (40–205 d). Table 2 lists the EIS fitting parameters of the T700/VE composite specimen.

**Figure 4:**
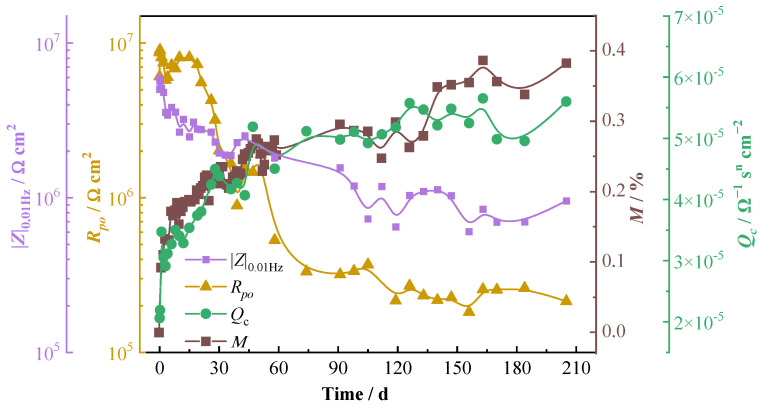
Variations of the percentage mass change, resin capacitance and resistance, and low-frequency impedance of T700/VE specimen with time.

Usually, the changes in the capacitance and resistance of polymer material can reflect the permeation of electrolytes within the polymer resin and the changes in the polymer’s barrier property. Figure 4 shows the variations of *Q*_c_ and *R*_po_ of the resin matrix as a function of time. It can be seen that the *Q*_c_ increases rapidly during the initial immersion and then gradually increases, while the *R*_po_ decreases at first and then remains relatively stable after 120 d of immersion. The percentage mass change (*M*/%) of the composite with time obtained by the weight gain test is also presented in Figure 4. It is seen that the variation trend of the *M* is in good consistent with that of the *Q*_c_, demonstrating a fast increase in the moisture uptake during the first 6 d immersion and then a gradually decreasing absorption rate with time. It can be noticed that the percentage mass change of the composite in 205 d of immersion is very small—less than 0.4% of the original value of the specimen. This value is very close to the reported data for carbon fiber-reinforced vinyl ester composites in seawater in the literature [7,35]. This again demonstrates that the vinyl ester-based composites possess very good stability and durability in salt water. A comparison among the mass change (*M*/%), the capacitance element (*Q*_c_), and the pore resistance (*R*_po_) show that the variation tendency of the *M* with time is well consistent with that of the *Q*_c_ and converse with that of the *R*_po_. This is because the dielectric constant of water (76.6−80.2) is much bigger than those of polymers (2.1−12.2) [14]. The water uptake of the polymer resin leads to an increase in the permittivity of the composite material, and therefore, the capacitance would be increased [36]. Meanwhile, the water uptake increases the ion conductivity of the material and thereby decreases the resistance. Comparing the variations of the *Q*_c_ and the *R*_po_, it can be noticed that the variation amplitude of *Q*_c_ is less than half order of magnitude (from 2.06 × 10^−5^ Ω^−1^ s^n^ cm^−2^ to 4.61 × 10^−5^ Ω^−1^ s^n^ cm^−2^), while the variation amplitude of *R*_po_ is more than one and a half orders of magnitude (from 6.09 × 10^6^ to 1.30 × 10^5^ Ω cm^2^), which is much bigger than the variation amplitude of *Q*_c_. So, the *R*_po_ might be a more suitable parameter to evaluate the performance of the composites, which changes more sensitively with water absorption. This is consistent with the literature [16], in which the authors thought that the pore resistance could possibly be a measure of the physical damage to the carbon fiber-reinforced composite.

The impedance |*Z*|_0.01Hz_ is a parameter, which can be obtained without fitting and analyzing the impedance spectra, so it is often used to estimate the barrier property of coating materials instead of the resistance *R*_po_. The curve of |*Z*|_0.01Hz_ with immersion time is also presented in Figure 4. It can be seen that though the value of |*Z*|_0.01Hz_ is lower than that of the *R*_po_ in the beginning and becomes higher in the later immersion period, it is obvious that, similar to the *R*_po_, the variation trend of |*Z*|_0.01Hz_ is also converse with that of the mass change (*M*/%). When the water absorption percentage of the composite increases rapidly, the value of |*Z*|_0.01Hz_ decreases quickly, and when the variation of the water absorption becomes smaller, the decreasing rate of the |*Z*|_0.01Hz_ becomes slower. Thus, there is some correlation between the |*Z*|_0.01Hz_ and the performance change of the carbon fiber-reinforced composites.

### 3.2. SEM-EDS and Bending Test Results of 700/VE Composite

**Figure 5:**
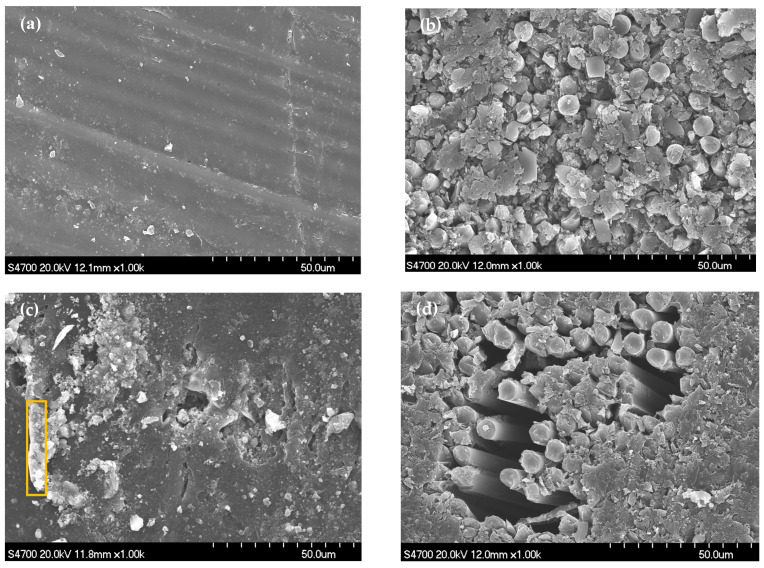
SEM images of the 700/VE specimen. (**a**,**b**) before immersion; (**c**,**d**) 205 d of immersion.

Figure 5 shows the surface and cross-section morphologies of the 700/VE composite before and after immersion in 3.5% NaCl solution by SEM measurement. Before immersion, the surface is clean (Figure 5a), and the interface between the carbon fiber and resin matrix is intact (Figure 5b). The main elements detected by EDS are C (86.05 wt%), O (6.26 wt%), and N (7.33 wt%). After 205 d of immersion, most areas of the composite maintain integrity in microstructure; only signs of slight damage were observed in a few local areas, which is manifested by the dissolution of some polymer resin and interfacial regions broken between fibers and resin (Figure 5c,d). It also can be noticed that some white precipitates are covered on the surface of the specimen. The composition analysis of the white precipitates (shown in yellow square in Figure 5c) shows that except for the C (51.36 wt%), O (13.35 wt%), and N (0.89 wt%) elements, there are a certain amount of Na (10.34 wt%), Cl (10.52 wt%), and small amounts of Ca (4.99 wt%), Fe (3.46 wt%), K (1.56 wt%), Mg (1.34 wt%), and Si (0.73 wt%) detected. This suggests that the precipitates may come from solution precipitation or the degradation product of the resin.

Table 3 shows the flexural modulus (*E*_f_) results obtained from four-point bending tests for the T700/VE composite specimens before and after immersion in 3.5% NaCl solution at different times. From 0−60 d, the values of *E*_f_ experience a slight increase, but the overall changes are very small. However, as the electrolyte further penetrates into the composite, the value of *E*_f_ decreases significantly, which is 129 GPa and 123 GPa after 130 d and 205 d of immersion, respectively. Compared with the initial value of 740 GPa, the decreasing amplitude of the *E*_f_ is 82.5% and 83.4%, respectively. This is because that after water is absorbed, the polymer matrix becomes softer, which will cause debonding to occur at the fiber–matrix interface, therefore decreasing the adhesion force [2,8]. The fibers are not able to contribute well to the load transfer between the fibers and the matrix, therefore resulting in a decrease in the bending property of specimens.

Table 3 also shows the value and the reduction of the |*Z*|_0.01Hz_ at the same time as *E*_f_ measured. Comparing with the results of the EIS and bending tests, it is noted that the flexural modulus of the T700/VE specimen experiences no big change before 60 d but a significant decrease after 205 d of long-time immersion. The |*Z*|_0.01Hz_ curve in Figure 4 shows a fast decrease at first and then towards a stable state after about 90 d. The decreasing magnitude of the |*Z*|_0.01Hz_ at 130 d and 205 d is 80.7% and 83.2%, respectively, which is very close to that of flexural modulus (82.5% and 83.4%) at the same time. The data in Table 3 also show that compared with the four-point bending test, the EIS test is a more sensitive method for evaluating the performance of the carbon fiber-reinforced composite, especially in the early stage because the bending test is a destructive method and the dispersion of the data is relatively large. So, as a non-destructive method, EIS technology is more suitable for evaluating the degradation and performance of carbon fiber-reinforced composite materials. It is worth mentioning that there might be some relationship existing between the results of the bending test and the EIS test, which needs more study to be carried out in future work.

### 3.3. Evaluation of Degradation Performance of T700/VE Composites by Phase Angles in Middle Frequency

**Figure 6:**
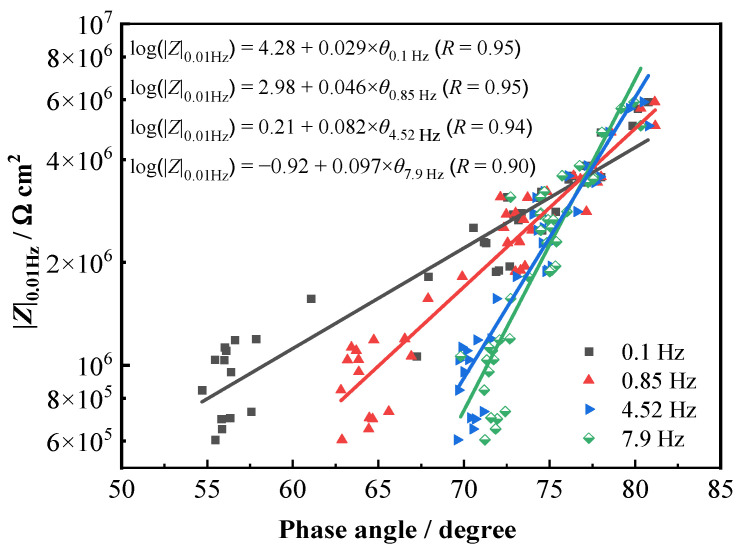
Fitting results of |*Z*|_0.01 Hz_ and several phase angles in middle-frequency range for T700/VE composite.

Our previous work suggested that the measurement of the phase angles in the middle−frequency range can be used to quickly evaluate the protection performance of organic coating on the metal substrate because a high correlation is found between the phase angles in the middle range and the impedance |*Z*|_0.01Hz_ [26,27]. Since the phase angle is obtained in the middle-frequency range, it takes a very short time when measuring and is very suitable for rapid detection and evaluation in field application. Pearson’s correlation coefficient (*r*) is often used to evaluate the strength of a linear relationship between two variables. The closer the coefficient is to +1 or −1, the stronger the linear correlation. A positive value denotes a positive linear correlation and a negative value negative linear correlation. It is generally considered that *r* ≥ 0.7 represents a strong correlation, and *r* ≥ 0.9 is a very strong correlation [27,37,38]. In this study, Pearson correlation analysis was utilized to analyze the correlation between the variation of phase angles at different frequencies and that of the |*Z*|_0.01Hz_ with time for the T700/VE composite. Table 4 presents the obtained *r* values in a certain frequency range. It was found that in the frequency range of 0.01–23.9 Hz, the values are higher than 0.75, which demonstrates that a strong correlation exists between the variation of phase angle in 0.01–23.9 Hz and the |*Z*|_0.01Hz_. The phase angles in the frequency range (0.1 Hz, 0.85 Hz, 4.52 Hz, and 7.9 Hz) that the *r* higher than 0.9 was selected and the linear relation curve between phase angles and the |*Z*|_0.01Hz_ was fitted by the Origin software. The results are shown in Figure 6, which denotes a very good linear relationship between the phase angle at each frequency selected with the logarithm of |*Z*|_0.01Hz_. Since measuring in certain middle-frequency domains takes a much shorter time compared with that in low-frequency domains, it is suitable for fast testing and evaluating for field applications. So, for the T700/VE carbon fiber-reinforced polymer composite, the phase angles in a certain middle-frequency range, such as 1 to 10 Hz, may be used to rapidly detect and evaluate its performance and degradation, just like for evaluating the organic coatings performance [26,27].

**Figure 7:**
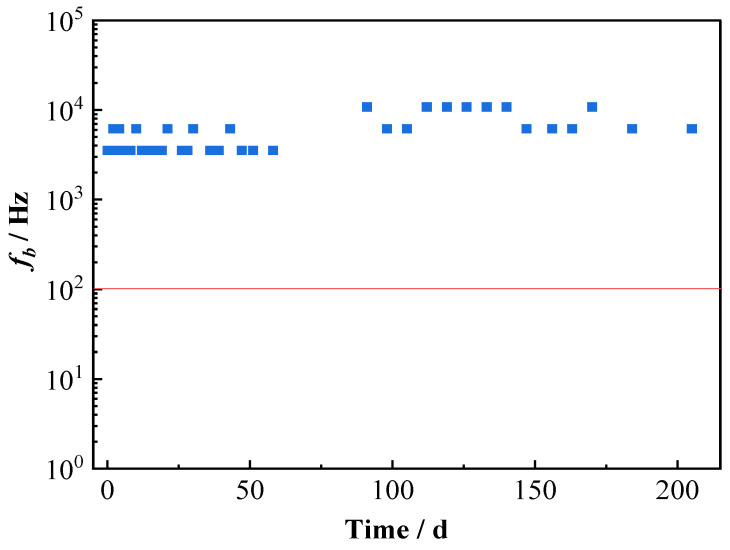
Variation of *f*_b_ with time for T700/VE composite specimen.

**Figure 8:**
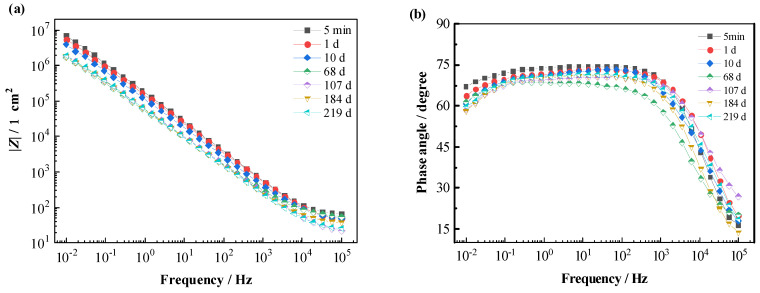
EIS Bode spectra of T700/BMI composite in 3.5% NaCl solution. (**a**) Impedance modulus; (**b**) Phase angle.

The breakpoint frequency (*f*_b_) was extracted from the Bode phase plot in Figure 2, and the variation of *f*_b_ with time is shown in Figure 7. It can be seen that *f*_b_ changes slightly with the testing time, fluctuating mainly in the range of 3.5 × 10^3^ Hz and 1.1 × 10^4^ Hz. It was reported in the literature that, for the polymer-coated metal system with coating degradation, the *f*_b_ shifts to larger values. When it reaches a value higher than 10^2^ Hz (marked as the red horizontal line in Figure 7), visual corrosion occurs under the organic coating [22,25]. However, the result in Figure 8 indicates that for the studied T700/VE composite, the value of *f*_b_ varies with no big change and keeps higher than 10^2^ Hz during the whole immersion period. This means that the *f*_b_ method is not suitable for monitoring the degradation of carbon fiber-reinforced polymer composite, which is consistent with the report by Alias et al. in the study of two other carbon fiber composites [15]. For the metals coated with organic coatings, in the degradation process of organic coatings, a good correlation exists between the *f*_b_ frequency in the high-frequency domain and the coating delamination area on metals; therefore, the degree of the coating deterioration can be obtained from measuring the *f*_b_ frequency. In the Bode phase plot, the phase angle maxima usually shift to higher frequencies with testing time for the coated metals. However, it is noticed that in the Bode phase plot of the T700/VE composite (Figure 2), there is almost no obvious change in the high-frequency range, probably because of its lack of micro-cracks as continuous diffusion channels through the thickness of the specimen as an organic coating [36]. So, the *f*_b_ method is not suitable for monitoring the degradation of carbon fiber-reinforced polymer composites.

### 3.4. EIS spectra of T700/BMI Composite and Degradation Performance Evaluation by Phase Angles in Middle Frequency

Figure 8 shows the Bode spectra of the T700/BMI composite immersion in 3.5% NaCl solution. Similar to the spectra of the T700/VE specimen, the spectra also present no big change after a long time. At 219 d of immersion, the value of |*Z*|_0.01Hz_ reaches 1.9 × 10^6^ Ω cm^2^ with a decrease of half order of magnitude compared with the initial value (7.1 × 10^6^ Ω cm^2^), and the phase angle at 0.01 Hz frequency is as high as 60 degrees. These demonstrate that the T700/BMI composite has very good durability in 3.5% NaCl solution under open circuit potential conditions.

**Figure 9:**
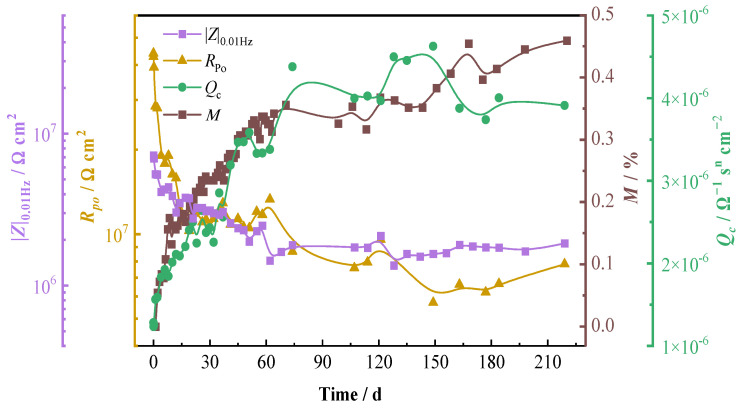
Variations of the percentage mass change, resin capacitance and resistance, and low-frequency impedance of T700/ BMI specimen with time.

The impedance data in Figure 8 were fitted by the equivalent circuit models in Figure 3, and the EIS fitting parameters are shown in Table 5. The changes of the constant phase angle element *Q*_po_ and the pore resistance *R*_po_ with the time of the T700/BMI composite are presented in Figure 9, in which the impedance |*Z*|_0.01Hz_ and the percentage mass change (*M*/%_t_) of the composite by weight gain test are also shown. Similar to the T700/VE composite, the *M*/% of the T700/BMI composite after 219 d of immersion is also very small, which is about 0.45% of the original value. It can be seen that the variation tendency of *M*/% with time is well consistent with that of the capacitance element *Q*_po_ and converse with that of the resistance *R*_po_ and impedance |*Z*|_0.01Hz_. This verifies that the behavior of EIS electrochemical parameters, such as *Q*_po_, *R*_po_, and |*Z*|_0.01Hz_, has a good relationship with that of the water adsorption for the T700/BMI composite. This is in good consistency with the results of the T700/VE composite.

**Figure 10:**
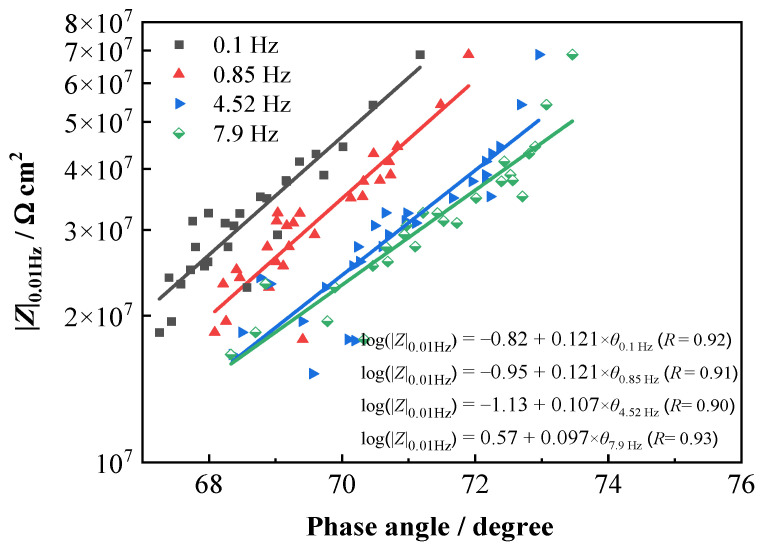
Fitting results of |*Z*|_0.01Hz_ and several phase angles in middle-frequency range for T700/BMI specimen.

The correlation between the variation of phase angles at various frequencies and the |*Z*|_0.01Hz_ with testing time for the T700/5428 composite was analyzed by Pearson’s correlation coefficient (*r*) too. Table 6 presents the obtained *r* values. Similar to the above result of the T700/VE composite, a strong correlation (*r* > 0.7) was found to exist between the variation of phase angle in the 0.01–23.9 Hz frequency range and that of the |*Z*|_0.01Hz_. Figure 10 shows the fitting results of the linear relation curve between the |*Z*|_0.01Hz_ and the phase angles in 0.1–10 Hz frequency (0.1 Hz, 0.85 Hz, 4.52 Hz, and 7.9 Hz) by the Origin software. It is clear that a very good linear relationship exists between the logarithm of |*Z*|_0.01Hz_ and the phase angles. The results of the T700/5428 composite are in good agreement with those of the T700/VE composite, confirming again that the values of phase angles in a certain middle frequency may be suggested to monitor the degradation of carbon fiber-reinforced polymer composites in engineering applications.

**Figure 11:**
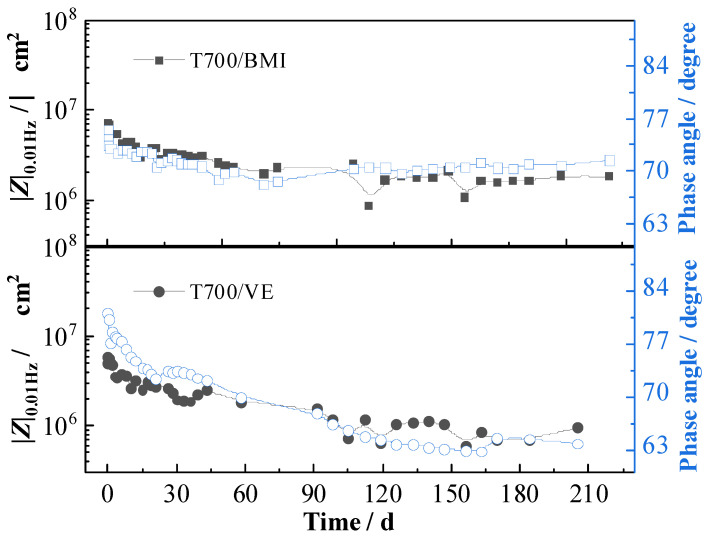
Variations of phase angle at 10 Hz and |*Z*|_0.01Hz_ with testing time for two composites.

Because the measurement in lower frequency takes longer time than that in higher frequency, in terms of the need for rapid detection in field applications, it is suggested to use that testing of the phase angle around 10 Hz (*θ*_10Hz_) to evaluate the performance of the carbon fiber-reinforced polymer composites instead of testing the low-frequency impedance (|*Z*|_0.01Hz_). Figure 11 shows the variations of |*Z*|_0.01Hz_ and the phase angle at 10 Hz (*θ*_10Hz_) with testing time for two carbon fiber-reinforced polymer composites. For each composite material, the curve of *θ*_10Hz_ shows a very similar decreasing tendency to that of the |*Z*|_0.01Hz_. That is, the phase angle at 10 Hz (*θ*_10Hz_) shows a high correlation with the |*Z*|_0.01Hz_. Therefore, *θ*_10Hz_ could be suggested to quickly detect and evaluate the performance of carbon fiber-reinforced polymer composites in 3.5% NaCl immersion.

## 4. Conclusions

The performance of T700/VE and T700/BMI carbon fiber-reinforced polymer composites in 3.5% NaCl solution for long-time immersion was investigated by the electrochemical impedance spectroscopy, weight gain test, combined with bending test and scanning electron microscope. Both composites have very good chemical stability under the natural immersion state, manifesting no big change in the EIS spectra, a small amount of water uptake (percentage mass change (*M*/%) less than 0.5%), and good integrity in microstructure under electron microscope observation. However, the slight damage to the fiber/resin interface in local areas results in a significant decrease on the flexural modulus (around 84%). The variation of the moisture absorption percentage by weight gain test shows good consistency with that of the resin capacitance (*Q*_c_), the resin resistance (*R*_po_), and the low-frequency impedance (|*Z*|_0.01Hz_). The measurement of the phase angles in the middle-frequency range was first applied to evaluate the performance of carbon fiber-reinforced polymer composites. There is a good linear relationship between the variations of phase angles in 0.1–10 Hz frequency range and the |*Z*|_0.01Hz_. In terms of the need for rapid testing in field applications, it is suggested that the phase angle at 10 Hz (*θ*_10Hz_) might be suitable to evaluate the performance of the composites instead of the low-frequency impedance, which also needs further verification in more composites materials in future work. The *f*_b_ method is not suitable for monitoring the degradation of carbon fiber-reinforced polymer composites. The traceability relationship between the bending test and EIS test results needs more study to be carried out in future work.

## Figures and Tables

**Table 1:** The composition of the carbon fiber reinforced composite materials.

Sample	Matrix	Reinforcement and Volume Fraction
CF/VE	Vinyl ester resin	T700 carbon fiber (60%)
CF/BMI	5428 Bismaleimide resin	T700 carbon fiber (55%)

**Table 2:** The impedance fitting parameters of the T700/VE composite.

Time	*R*_s_ (Ω cm^2^)	*R*_po_ (Ω cm^2^)	*Q*_c_ (Ω^−1^ s^n^ cm^−2^)	*n*_Qc_	*R*_ct_ (Ω cm^2^)	*Q*_dl_ (Ω^−1^ s^n^ cm^−2^)	*n*_Qdl_
5 min	4.177	6.09 × 10^6^	2.06 × 10^−5^	0.8990			
5 h	4.209	1.30 × 10^7^	2.24 × 10^−5^	0.8889			
10 d	3.128	8.09 × 10^6^	3.43 × 10^−5^	0.8444			
30 d	2.886	2.01 × 10^6^	4.38 × 10^−5^	0.8375			
43 d	2.267	1.56 × 10^6^	4.05 × 10^−5^	0.8457	9.19 × 10^6^	6.87 × 10^−6^	0.4170
58 d	3.277	3.41 × 10^5^	4.57 × 10^−5^	0.8292	1.99 × 10^6^	7.61 × 10^−6^	0.8504
91 d	1.593	2.87 × 10^5^	5.08 × 10^−5^	0.8337	2.48 × 10^5^	4.44 × 10^−5^	0.8543
133 d	1.655	1.07 × 10^5^	6.09 × 10^−5^	0.8113	2.05 × 10^5^	8.93 × 10^−5^	0.8999
163 d	2.586	1.20 × 10^5^	6.38 × 10^−5^	0.7900	2.22 × 10^5^	3.56 × 10^−5^	0.8129
205 d	1.962	1.30 × 10^5^	4.61 × 10^−5^	0.8437	5.37 × 10^5^	4.04 × 10^−5^	0.6064

**Table 3:** *E*_f_ results of T700/VE composite specimens after immersion at different times.

	Before Immersion	30 d	60 d	130 d	205 d
*E*_f_/GPa	740	764	754	129	123
Reduction of E_f_		−3.3%	−1.9%	82.5%	83.4%
|*Z*|_0.01Hz_/Ω cm^2^	5.7 × 10^6^	1.9 × 10^6^	1.8 × 10^6^	1.1 × 10^6^	9.6 × 10^5^
Reduction of |*Z*|_0.01Hz_		66.7%	68.4%	80.7%	83.2%

**Table 4:** Pearson’s correlation coefficient (*r*) between the phase angles and |*Z*|_0.01Hz_ for T700/VE.

Frequency/Hz	0.01	0.1	0.85	4.52	7.9	13.7	23.9	127	1172	10,826
*r*	0.88	0.95	0.95	0.94	0.90	0.86	0.75	0.10	−0.31	−0.6

**Table 5:** The impedance fitting parameters of the T700/BMI composite.

Time	*R*_s_ (Ω cm^2^)	*R*_po_ (Ω cm^2^)	*Q*_c_ (Ω^−1^ s^n^ cm^−2^)	*n*_Qc_	*R*_ct_ (Ω cm^2^)	*Q*_dl_ (Ω^−1^ s^n^ cm^−2^)	*n*_Qdl_
5 min	59.52	4.40 × 10^7^	1.28 × 10^−6^	0.8259			
1 d	41.51	2.85 × 10^7^	1.56 × 10^−6^	0.8074			
10 d	44.17	1.63 × 10^7^	2.11 × 10^−6^	0.8014			
30 d	87.7	1.06 × 10^7^	2.43 × 10^−6^	0.7924			
37 d	66.22	1.19 × 10^7^	2.56 × 10^−6^	0.7983	4.05 × 10^7^	2.61 × 10^−7^	0.8309
68 d	52.07	1.50 × 10^7^	4.57 × 10^−6^	0.7574	8.26 × 10^6^	4.10 × 10^−6^	0.7612
107 d	17.84	6.90 × 10^6^	4.00 × 10^−6^	0.7825	4.77 × 10^6^	5.93 × 10^−6^	0.8090
149 d	24.42	5.71 × 10^6^	4.63 × 10^−6^	0.7718	1.17 × 10^6^	4.55 × 10^−6^	0.7969
184 d	35.08	5.74 × 10^6^	4.00 × 10^−6^	0.7911	1.16 × 10^6^	6.68 × 10^−6^	0.9584
219 d	22.76	7.04 × 10^6^	3.91 × 10^−6^	0.7940	1.65 × 10^6^	6.76 × 10^−6^	0.8740

**Table 6:** Pearson’s correlation coefficient (*r*) between the phase angles and |*Z*|_0.01Hz_ for T700/BMI.

Frequency/Hz	0.01	0.1	0.85	4.52	7.9	13.7	23.9	127	1172	10,826
*r*	0.92	0.89	0.95	0.94	0.91	0.86	0.78	0.40	0.08	−0.06

## Data Availability

Not applicable.
